# Caring for Dying People: Attitudes Among Iranian and Swedish Nursing Students

**DOI:** 10.4103/0973-1075.73643

**Published:** 2010

**Authors:** Sedigheh Iranmanesh, Karin Axelsson, Terttu Häggström, Stefan Sävenstedt

**Affiliations:** Razi Faculty of Nursing and Midwifery, Kerman Medical University, Kerman, Iran; 1 Department of Health Science, Lulea University of Technology, Luleå, Sweden

**Keywords:** Attitude toward caring for dying persons, Attitude toward death, Nursing students, Palliative care, Transcultural nursing

## Abstract

**Aim:**

To compare the attitudes of Iranian and Swedish nursing students toward caring for dying persons.

**Materials and Methods:**

Their attitudes were measured with the Frommelt’s Attitude Toward Caring of the Dying and the Death Attitude Profile Revised.

**Results:**

The results indicated that the participating Iranian students were more afraid of death and less likely to give care to dying persons than the Swedish participants.

**Conclusion:**

It is suggested that theoretical education should be individualized and culturally sensitive in order to positively influence the students’ attitudes, and promote professional development.

## INTRODUCTION

Palliative care is described as a way to meet the physical, mental, and spiritual needs of people who are chronically ill and dying.[1] Providing good palliative care requires an inner commitment from the professionals who are involved. The commitment depends on how these professionals view death and persons who are dying. Our view on caring for a dying person could be described by our attitude — a multifaceted concept — that includes views, perceptions, and values on many aspects of private and professional life. In this article, we use a definition by Eagly and Chaiken.[2] According to them, attitude is a psychological tendency expressed by evaluating a particular entity with some degree of favor or disfavor. The nurses’ attitudes toward caring for dying persons are reflected in their personal motivation, and manifested in their interaction with people who are at the end of their lives. This basic relationship is described by Mok and Chiu.[3] In their report, people who had reached the end of their lives expressed how the nurses’ love for them was visible through their use of soft voices and the time they spent to listen to them.

Leininger[4] suggests that all around the world, care is culturally constituted, and Holloway[5] states that nurses’ beliefs and values surrounding death can influence their relationship with people who are at the end of their life. Studies on the attitude of nursing students toward caring for people who are dying also indicate that cultural heritage can be translated into different ways of interactions with people who are dying.[6] Researchers have also described the effects of individual characteristics like age[7] and level of education[8] on the attitude of nursing students toward death, and caring for people at the end of life.

It seems that Iranian and Swedish nursing students have different attitudes toward death and caring for dying patients. There is no study that examines how the different cultural and social contexts of Iranian Nursing Students (INS) and Swedish Nursing Students (SNS) are reflected in their attitudes of caring for persons at the end of life. Such knowledge may contribute toward increasing the understanding of how to improve the education of nurses, to prepare them for giving good palliative care in both cultural contexts. The purpose of this descriptive study is to compare the attitudes of INS and the SNS toward caring for dying people.

### Context

The sample of nursing students in this study comes from two different cultural contexts. Sweden is a part of the European culture, whereas, Iran is a part of the Middle Eastern culture. Most religions are represented in both countries, but the majority of the people in Iran follow Islam, and those in Sweden follow Christianity. The two countries have different demographic indicators [Table 1], and it can be assumed that the average Iranian, compared to the average Swede, is more familiar with death in everyday life. The adult mortality rate in Iran is higher than that in Sweden.[9] The people of Iran have, in the last 30 years, experienced major wars and extensive natural disasters such as the Bam earthquake, which resulted in the death of a huge number of people. Although there is a difference in the experience of facing sudden death among Iranian and Swedish people, the latter have also experienced collective death such as in the Estonia ferry accident (year 1994) and the Asian tsunami (year 1995). In addition, some residents of Sweden have escaped wars and natural disasters in other countries.

**Table 1:** Health and welfare indicators in Iran and Sweden (WHO, 2004)

Country	Total population (millions)	Life expectancy in years for Males / Females	Child mortality rate among Males / Females*	Adult mortality rate among Males / Females**	Total health expenditure as % of GDP
Iran	69	56.1 / 59.1	39 / 36	190 / 118	6.5
Sweden	9	71.9 / 74.8	4 / 3	82 / 51	9.4

* = Under five years, (per 1000 population);

** = between 15 and 60 years, (per 1000 population)

Religion as one aspect of culture differs not only in different dominating religions, but also in how dominating the religion is in the everyday life of people. Allport and Ross[10] claimed that there are two types of religiosity; intrinsic and extrinsic religiosity. They go on to state that, “the extrinsically motivated person uses his religion, whereas, the intrinsically motivated lives his religion” (p.434). These two types of religiosity are measured on the Religious Orientation Inventory (ROI). Extrinsic religiosity refers to church attendance, volunteer activities related to religion, and religious activities involving at least two people. Intrinsic religiosity refers to solitary activities such as praying, Bible reading, and personal belief in God. Signifiers such as, ‘religious,’ ‘secular,’ or even ‘God’ have dramatically different meanings and connotations in different cultures.[11] They are laden with historical, political, social, and theological implications that are unique to every given country and the subcultures therein. Iran is ruled by the authority’s theocrats and is regarded as a religious country, while Sweden is regarded as a secular country.[12,13] The majority (99.4%) of the people in Iran consider themselves as religious, but there is a low attendance (27%) in religious service.[14] In contrast, among Swedish people, 46% claim that they believe in a personal God,[15] and less than 10% attend religious services at least several times a month.[16]

Iran is in the group of countries where the nuclear family, based on marriage, prevails. The relations and sentiments among Iranian families are strong so that apart from the patient, the family members will be severely struck if they are informed that their loved one is close to death.[17] These reactions, which make the patient worse or the family disrupted, may lead the nurses to give them care on the one hand and support them emotionally, but on the other hand, it would be unlikely that the nurses could talk to them about death, give them an honest answer about the patient’s condition, or even to involve them in their care. In contrast, Swedes often practice nonfamily living in young adulthood, leaving the parental home before entering into a co-residential partnership through cohabitation or marriage.[18]

There is also a difference in the inclusion of the subjects about death and palliative care in the national curriculum between the two countries. In Iran the overall national curriculum for registered nursing education includes just a few hours of academic education about death. Cheraghi, Payne, and Salsali[19] have also stated that there are no hospice care units in Iran like those that are common in the Western countries. Palliative care, including hospice care, is well-established in Sweden, where universities provide special training courses in palliative nursing, and support the quality and development of palliative care through teaching and research.[20] In addition, each student receives personal supervision during their practical education.

## MATERIALS AND METHODS

The ethical implications of the study were tried and approved by the Heads of the Departments of the Nursing Students’ Education from both universities, prior to the collection of data. The study employed a descriptive, explorative design, and was conducted in the Tehran University, College of Nursing, in Iran and Luleå University of Technology, Department of Health Science, in Sweden. A specially designed questionnaire to obtain the background information and two validated instruments were used to obtain data in the two countries. The three questionnaires used were in English.

### Background information

First, a questionnaire was designed to obtain the background information, which was assumed to influence the attitude toward caring for dying persons. It was based on the experiences of a pretest among students in both countries. It included questions about gender, age, religion, intrinsic and extrinsic religiosity, previous experience of the death of someone close, earlier experiences of life-threatening situations such as severe diseases, and the present residential situation.

### The instruments

To measure the degree to which nursing students considered themselves as likely or unlikely to care for people at the end of life, Frommelt’s Attitude toward Caring of the Dying (FATCOD)[21] was used. This scale consisted of 30 items designed to measure the participants’ attitudes toward providing care to dying persons. The questions were graded from one to five (1 = strongly disagree – 5 = strongly agree). Fifteen of the items were worded positively and fifteen were worded negatively.

The extent to which nursing students favored or disfavored the statements about death was measured with an instrument named Death Attitude Profile Revised (DAP-R).[22] This was a multidimensional measurement using a seven-point Likert-type scale. It was composed of 32 questions that described the attitudes toward death. A factor analysis made by Wong *et al*.[22] on the research carried out in an American setting revealed that the questions could be divided into five components including fear of death (7 items), death avoidance (5 items), neutral acceptance (5 items), approach acceptance (10 items), and escape acceptance (5 items). The questions were graded from one to seven (1 = strongly disagree – 7 = strongly agree).

The instruments FATCOD and DAP-R have been used by several researchers[8,23–25] to examine the attitude toward death/ or attitude toward caring for dying people. Payne, Dean, and Kalus[26] also used the DAP-R scale to compare the levels of death anxiety and coping responses in palliative care and accident and emergency nurses.

These two scales were originally developed and tested in an American cultural context, which was different from both the Iranian and the Swedish cultural contexts. The validity of both the scales had been assessed through a content validity discussion in both countries. Scholars of nursing care at the College of Nursing, University of Tehran, and the Department of Health Science, Luleå Technical University had reviewed the content of the scales. They had agreed upon a reasonable representation of the questions on the scales of the universe of religious and cultural aspects of death and dying in Iran and Sweden.

### Data collection and analysis

The first author administered the data collection using the same procedure in both the countries. All the nursing students in semesters four, five, and six, at both the universities, were invited to participate during the spring of 2007. All students had an experience of dealing with dying patients. The students were approached in classroom settings during regular university hours. They were informed in their own language both verbally and with written information about the study, its purpose, and method, and were also informed that the participation was voluntary. They were also told not to state any name or other personal information on the questionnaires in order to secure confidentiality, so that no single student could be identified in the data or in the final report.

In Iran, 120 sets of questionnaires were distributed, with a drop-out of ten. In Sweden, 114 sets of questionnaires were distributed, with only one drop-out. In all the collected data, 99% of all the questions were answered in both countries. Data from the questionnaires were analyzed using the Statistical Package for Social Scientists (SPSS). A Kolmogorov-Smirnov test indicated that the data were sampled from a population with normal distribution. The comparison between the two groups, in all the measured factors, was done using a descriptive analysis, and an independent t-test. Using a cross table with a chi-square test and correlation of the factors, including FATCOD’S indicators, the demographic variables expected to influence the attitude toward caring for dying people were identified. To facilitate the analysis, the answers of the questions with more than two answering alternatives were regrouped. In the FATCOD-scale, the alternatives ‘agree’ and ‘strongly agree’ as well as ‘disagree’ and ‘strongly disagree’ were grouped together in a single category, and five levels were reduced to three levels. The same approach was used for the DAP-R sub scales, where the seven levels were reduced to three levels. In cross tables, where the chi-square test indicated significant differences, the data were also tested for correlation using the Spearman’s test. The significance level was set at *P* < 0.05.

### Sample

A convenience sample of 223 nursing students from Iran and Sweden were gathered, of which 110 were INS and 113 were SNS. The size of the sample was selected based on previous published research using the same instruments and was assumed to be sufficient to make a comparison between the two subgroups. A descriptive analysis of the background information revealed that about 80% of the INS and 98% of the SNS were females. All the INS belonged to the age group of 20 – 29 years (mean = 25), while their Swedish peers were less homogenous. They varied from 20 – 50 years (mean = 35). The students also differed in religion and religiosity. Almost all the INS professed to Islam (99%), and all of them stated that they believed in God and took part in religious activities every day or several times a week. In contrast, 56.6% of the SNS claimed that they were Christians, but only 10% indicated that they took part in religious activities, and 22% indicated that they had an intrinsic religiosity. Among the INS, 93% were living with their parents, whereas, more than 95% of the SNS were living alone or with a partner [Table 2].

**Table 2:** Background information on Iranian and Swedish nursing students

Characteristics	Iranian nursing students	Swedish nursing students
Mean age in years	25 (range 20 – 29)	35 (range 20 – 50)
Religion	99% (Islam)	56.6% (Christianity)
No religion	0	38.7%
Other religion	1%	3.6%
Religious activity		
Every day or some times a week	92%	10%
A few times per month	3%	8%
A few times per year or never	5%	82%
Belief in God		
Believer	100%	22%
Nonbeliever or unsure	0	78%
Residential situation		
Living with parents	93%	3%
Living with partner	4%	60%
Living with others or alone	3%	37%
Education on hours of death and dying	2 – 4	40

## RESULTS

### Validity and reliability

To examine the context validity of the five identified components of the DAP-R scale, a factor analysis (Rotated Component Matrix) on the results from the INS and the SNS was performed. The loading of the items was similar to the American results, except for the component of fear of death, where the loading for item numbers 21 and 32 was lower than that in the American results (0.31 vs. 0.75). The reliability of both the scales was tested by assessing the internal consistency. The internal consistency for both the scales in both the groups was relatively good (Cronbach’s alpha for FATCOD = 0.72; DAP-R = 0.68). All the informants’ comprehension of the English language in the questionnaires was checked by a question added to the last questionnaire. The comprehension was graded from 1 – 10. All the INS graded their comprehension from five to nine (mean = 7) versus from 7 – 10 (mean = 8.5) among SNS.

### Attitudes toward caring for dying persons

According to the independent t-test analysis [Table 3], there were both similarities and significant differences between the attitudes of the INS and the SNS toward palliative care. Both the groups had positive attitudes toward taking part in palliative care, but the INS were less likely than the SNS to interact with dying persons and talk to them about death.

**Table 3:** Comparison of means between the Swedish and Iranian nursing students, analyzed with T-test, for some selected indicators of the FATCOD scale

Questions	Swedish nursing students (T-test scores)	Iranian nursing students (T-test scores)	*P*-value
Negative attitude toward caring for dying people and their families	2.1	2.93	0.012
I would be uncomfortable talking about impending death with the dying persons	2.78	3.5	0.003
It is difficult to form a close relationship with the family of a dying person	2.24	3.24	0.004
I would not want to be assigned to care for a dying person	1.84	2.73	0.001
The length of time required to give nursing care to a dying person would frustrate me	2.26	3.14	0.000
The nurse should not be the one to talk about death with the dying person.	1.6	2.07	0.02
Positive attitude to caring for dying people	4.1	4.14	0.134
Nurses should permit dying people to have flexible visiting schedules	4.57	4.38	0.075
The dying person and his or her family should be the decision-makers	4.3	4.24	0.175
Dying people should be given honest answers about their conditions	3.97	3.77	0.125

The findings also indicated that attitudes toward death and some of the background information influenced their attitudes toward caring for people at the end of life. An independent *t*-test analysis indicated that the INS tended to have greater fear of death and were likely to view death as a gateway to the happy afterlife than the SNS. The Swedish informants viewed death as more natural compared to the Iranians.

According to the Cross-table analysis [Table 4], there were some factors influencing the attitude toward caring for dying people. For instance, fear of death and religiosity increased the negative feelings toward caring for people at the end of life. The students who reported that they were more religious and more likely to view death as a fearful event, had a more negative approach toward caring for persons at the end of life, than those who were less religious and less likely to view death as a fearful event. Age and a natural outlook toward death decreased the negative attitude toward caring for dying people. The students who were older and had a more natural view of death had a less negative attitude toward caring for people who were dying, than those who were younger and had a less natural view of death. There was no significant correlation between other factors and the FATCOD scores.

**Table 4:** Correlation between indicators expressing negative attitudes toward caring for dying people (FATCOD) and selected background variables, together with selected indicators of attitudes toward death (DAP-R scale) among both Swedish and Iranian nurse students

Negative indicators toward caring for dying people (FATCOD)	Age Spearman’s test / *P*-value	Intrinsic religiosity Spearman’s test / *P*-value	Extrinsic religiosity Spearman’s test / *P*-value	Death is no doubt a grim experience *** Spearman’s test / *P*-value	Death is a natural aspect of life **** Spearman’s test / *P*-value
I would be uncomfortable talking about impending death with the dying persons	r = - 0.221*P = 0.002		r = 0.196 *P* = 0.04	r = 0.177 *P* = 0.03	r = - 0.297*P = 0.003
It is difficult to form a close relationship with the family of a dying person	r = - 0.281*P = 0.003	r = 0.393**P = 0.001			r = - 0.285*P = 0.002
I would not want to be assigned to care for a dying person	r = - 0.305**P = 0.027		r = 0.591**P = 0.000		
The length of time required to give nursing care to a dying person would frustrate me	r = - 0.390**P = 0.004				

* Correlation is significant at the level of *P* < 0.05 (two-tailed);

** Correlation is significant at the level of *p* < 0.01(two-tailed);

*** One of the indicators measuring fear of death in DAP-R scale;

**** One of the indicators measuring natural acceptance of death in DAP-R scale

## DISCUSSION

This transcultural study assesses some influential factors in society that shape the nursing students’ attitudes toward death and dying in two different countries. The focus was not only on the differences that made the two groups distinctive, but also on the similarities that brought them together. The study revealed that the SNS were more likely to provide palliative care to people than the INS. These differences were correlated with some cultural, social, and individual factors, which were significantly different between these two groups. There were differences in the levels of religiosity, education about palliative care, age, and personal maturity, as well as their view regarding death. The results, based on their answers to the two questionnaires, indicated that both groups’ attitudes toward death influenced the way they related to people at the end of life. This result corresponded with the findings of earlier studies,[23,24] which reported a significant correlation between attitudes toward death and attitudes toward caring for people who were dying.

The findings indicated that the INS marked themselves as more religious and they tended to view death from a more religious perspective compared to the SNS. This was in line with Tomás-Sábado’s and Gόmez-Benito’s[27] statement that belief about what happened after death often was linked to religious issues. Religiosity in this study was found as a factor that increased negative attitudes toward caring for people who were dying. This finding was in contrast to an earlier study carried out in an oncology clinic in Sweden, where Lundmark[28] found a positive correlation between religiosity and a positive attitude toward giving spiritual care to the persons who were dying. The difference in the results indicated that religiosity was not the only influential factor for the nursing students’ attitudes toward caring for dying persons; other cultural and social circumstances could also be influential. The INS feared death more than the SNS, and hence, they were less likely to talk about death and interact with the dying people. One possible explanation for this finding could be that the INS, compared to their Swedish peers, were coming from a society with a greater exposure to death,[9] which made death a more visible threat to their own and their relatives’ lives. In contrast to the INS, the SNS were more likely to view death as a natural part of life. According to Rooda *et al*.,[23] the nurses who viewed death as a natural part of life tended to have a more positive attitude toward giving care to the people who were dying than nurses who did not share this view. It could be assumed that the students’ own fear of death affected their attitudes toward interacting with people who were dying. The assumption was supported by a study which showed that the nurses’ anxiety about death was manifested by the fact that they were reluctant to talk about death, think about it, or be with dying persons.[29]

On the other hand, according to the results, the SNS were older than the INS, which may have contributed to their being less fearful of death, and also being more likely to give care to dying people than their counterparts in Iran. This finding could be supported by earlier studies, which found age and psychosocial maturity to be inversely related to death anxiety,[30] and positively correlated with attitudes toward giving care to people at the end of life.[7,31]

Researchers[8,32] have reported that nursing education, including courses dealing with death and dying; have reduced nursing students’ anxieties concerning death. Nursing students who were more educated in palliative care had a more positive attitude when providing care to terminally ill persons, than those with a lower level of education. It is possible that lack of educational experience among the INS may have influenced their attitudes toward people who were dying. The subject about death and caring for people at the end of life has been included in the Swedish nursing curriculum. However, the Iranian healthcare system has presently begun to provide special care for people at the end of life, but in the nursing curriculum, only a few hours are devoted to educate the students about death and dying.

Despite the differences between the two groups in their attitudes toward caring for dying persons, there are some similar views on giving care to these people. Professional and personal competence was seen as important by both groups. It could be explained by the fact that the notion of death has its origin in a commonly held ‘truth’; in this case, the acknowledgment that our common humanity is affirmed in death and dying, which is an essential part of being human.[5]

### Limitations

Some limitations need to be addressed when evaluating the results of this study. Both the instruments used here were designed in an American cultural context, which is different from the cultural context of Iran and Sweden. Therefore, a content validation of the scales was done in both countries. It was found that cultural issues such as questions about religiosity were general and could be applied both in the Muslim and a Christian context.

All questions were posed in the English language, so the differences between the two groups in their comprehension of the questions could be viewed as a confounding variable, which may have contributed to the results, especially as the INS scored lower in their comprehension of the questions. In both the groups, the participants were encouraged to ask about the content if they did not understand a question.

The respondents were predominantly female, which limits the generalizability of the results for male students. An earlier study reported higher levels of empathy scores for females, which were associated with higher levels of death anxiety.[31] As this study was based on a convenient sample and the participation was voluntary, there might have been a selection bias that affected the possibility to generalize the results of all the INS and the SNS. However, clear differences between the two groups of students in attitudes toward death and caring for dying people indicated that the size of the sample was sufficient and that the results could be generalized to similar contexts in both countries. Further research is necessary to illuminate the influences of culture, education, and age/ maturation on the attitudes of caring for dying persons among nursing students.

## IMPLICATIONS

This study provides nurses and nurse educators with some valuable insights about how culture, religion, age, and education may influence nursing students’ attitudes toward palliative care. It is suggested that attitudes toward death are multidimensional and may vary in different cultural contexts. Programs aimed at raising the students’ self-awareness of their attitude, accompanied by interventions intended to decrease fear of death are important in educational programs. As revealed in this study, maturity and personal growth are some of the factors that affect students’ attitudes toward caring for people at the end of life. Students should gain experience in palliative care under well-educated preceptorship[33] during their education. Exposure to suitable narratives[34] under individual or group supervision during their clinical practice offers serious prospects for developing profound education, which at the same time can be seen as supporting essential, personal maturation. A positive and caring relationship can help to improve a person’s and his/ her relative’s emotional and physical well-being, and increase satisfaction with palliative care, as also the level of work satisfaction among the nurses.
